# One confirmed and one potential human case of influenza A(H5N1) detected through an expanded subtyping protocol

**DOI:** 10.1128/asmcr.00165-25

**Published:** 2025-12-10

**Authors:** Grant P. Higerd-Rusli, Abraar Karan, Seth A. Hoffman, Ingrid E. A. Morante, ChunHong Huang, Malaya K. Sahoo, Matthew M. Hernandez, Benjamin A. Pinsky

**Affiliations:** 1 Department of Pathology, Stanford University School of Medicine 10624 , Stanford, California, USA; 2 Division of Infectious Diseases and Geographic Medicine, Department of Medicine, Stanford University School of Medicine 10624 , Stanford, California, USA; 3 Clinical Virology Laboratory, Stanford Health Care, Stanford, California, USA; Rush University Medical Center, Chicago, Illinois, USA

**Keywords:** avian influenza, influenza, H5, HPAI

## Abstract

Current U.S. surveillance for highly pathogenic avian influenza A(H5N1) in humans prioritizes individuals with known animal exposures, potentially missing community-acquired infections. To address this gap, we implemented universal H5 subtyping of all influenza A-positive respiratory samples collected within our hospital system, regardless of patient exposure history. Between August 2024 and April 2025, we subtyped 4,488 influenza A-positive samples and identified two cases positive for H5 RNA in Alameda County, California, USA. The first case was a 14-month-old girl with mild respiratory symptoms and no H5N1 exposure risks; sequencing of the sample revealed an H5 gene closely related to clade 2.3.4.4b, genotype B3.13 viruses circulating in U.S. dairies. The second case was a 79-year-old male, also with no known exposures, whose sample reproducibly tested positive with a high cycle threshold value but could not be confirmed by public health laboratories. Both patients had evidence of co-infection with other common respiratory viruses. These findings, while requiring cautious interpretation due to low virus levels and the presence of potential confounding factors, highlight limitations in exposure-based testing and demonstrate the potential for cryptic H5N1 circulation. This report underscores that broader, geographically targeted surveillance may be a critical tool for early detection of potential community transmission of pandemic-capable pathogens.

## INTRODUCTION

Highly pathogenic avian influenza A H5N1 has spread globally in wild birds and led to outbreaks in increasingly diverse mammalian species (1). Since March 2024, an outbreak of H5N1 clade 2.3.4.4b has spread among dairy cows across the United States (1). Given that there is little to no pre-existing immunity in the human population, acquisition of efficient human-to-human transmissibility by H5N1 could lead to rapid global dissemination, resulting in substantial morbidity and mortality (2, 3). As of August 2025, 70 confirmed human cases have been detected in the United States, 65 of which have reported high-risk exposures to infected dairy cows or poultry and two of which reported exposure to backyard flocks, wild birds, or other mammals (4). There have been three reported infections in individuals without known exposures: one in Missouri, one in San Francisco, California, and another in British Columbia, Canada (1, 5, 6). There has been one fatal case in Louisiana (1).

Current influenza surveillance efforts in the United States include “routine surveillance,” where a finite subset of human influenza A-positive samples undergo additional testing (7). This may include H5 testing but only for samples without an identifiable seasonal influenza A subtype (8). As of 18 August 2025, routine surveillance demonstrated an H5 case positivity rate of ~0.003% (6/204,843) (4). Targeted subtyping for H5 is currently limited by strict testing criteria that prioritize individuals with known animal exposure (9, 10). Consequently, human cases, particularly those arising from potential human-to-human spread, may be missed. To evaluate whether cases may be going undiagnosed, we implemented universal H5 subtype screening of all influenza A-positive samples within our hospital system, regardless of patient exposure history. Here we report that this expanded screening identified two cases of H5N1 in individuals with no known H5N1 exposure risk in Alameda County, CA.

## MATERIALS AND METHODS

### H5 subtyping protocol

Nasopharyngeal samples that tested positive for influenza A, collected within the Stanford Health Care system, were subtyped for influenza A H5 using a previously described, multiplex, dual-target H5 RT-qPCR that includes primer probes for pan-influenza A (matrix) and an internal control (human RNaseP) (11). This assay was previously validated using synthesized nucleic acids and genomic RNA from cultured avian and human influenza A virus isolates. Using genomic RNA from Kilbourne F181, A/duck/Singapore/645/1997(H5N3) (BEI Resources NR-9682), the assay demonstrated a 95% lower limit of detection of 0.6 copies/µL for the H5 target and a 95% lower limit of detection of less than 0.5 copies per µL for the pan-influenza A matrix target. Given that the pan-influenza A target appeared more analytically sensitive than the H5 target, a pan-influenza A cycle threshold (C_T_) value of less than 40 cycles was required to interpret the H5 result. Samples that were positive for H5 were re-extracted and re-tested to confirm the result.

### Sequencing

Partial sequencing of the hemagglutinin (HA) gene was performed on the residual total nucleic acid eluates from Case 1. Four reverse transcription PCR (RT-PCR) reactions were performed on each eluate using the following H5 primer pairs: H5HA-205-227-For(2010) + FluA_H5_v4_2R (~1,070 base pairs [bp]), H5HA-205-227-For(2014) + FluA_H5_v4_2R (~1,070 bp), FluA_H5_v4_2F + FluA_H5_v4_1R (~584 bp), and H5HA-205-227-For(2010) + FluA_H5_v4_1R (~1,510 bp). These primers were previously published (11, 12). The primer sequences and final reaction concentrations are provided in Table 1.

**TABLE 1:** Primers used to generate amplicons for sequencing

Name	Sequence (5′−3′)	Final concentration (nM)
H5HA-205-227-For(2010)	CGATCTAAATGGAGTGAAGCCTC	400
H5HA-205-227-For(2014)	CGATCTTAATGGAGTGAAGCCCC	400
FluA_H5_v4_2R	AACTCCCTTCCAACTGCCTCAAA	400
FluA_H5_v4_2F	TGGGTACCATCATAGCAATGAGCA	400
FluA_H5_v4_1R	GTAACGACCCATTGGAGCACATCC	400

RT-PCR was performed on an Applied Biosystems Veriti thermal cycler using the Invitrogen Superscript III Platinum One-Step qRT-PCR kit (Thermo Fisher Scientific, Waltham, MA). Each 25 µL reaction contained 12.5 µL of 2× buffer, 5 µL nuclease-free water, 0.5 µL of enzyme mix, 1 µL forward primer, 1 µL reverse primer, and 5 µL of nucleic acids. Cycling conditions were as follows: 52°C for 60 min, 94°C for 2 min, then 45 cycles of 94°C for 15 s, 55°C for 30 s, and 68°C for 2 min; finally, hold for 10 min at 68°C.

The amplification products were individually processed for sequencing using enzymatic fragmentation and NEBNext Ultra II reagents (New England Biolabs, Ipswich, MA), as previously described (13). Libraries were sequenced on an Illumina MiSeq using single-end 150-cycle sequencing using the MiSeq Reagent Kit (version 3).

Reads were aligned to reference sequence EPI_ISL_19531296 from the Global Initiative on Sharing All Influenza Data (GISAID) using Burrows-Wheeler Aligner (version 0.7.12). Reads with a mapping quality score of <10 were discarded. Individual BAM files were generated using SAMtools (version 1.12). The BAM files from each sequencing library representing the eight amplicons (two eluates amplified with four primer pairs) were then merged, and the consensus sequence was generated using Genome Analysis ToolKit (version 4.2.0.0). Any position with <2 reads was labeled as N. Only the region with a coverage of >500× encompassing positions 1,121–1,701 was submitted to GISAID (accession no. EPI3684158, isolate ID: EPI_ISL_19597300) and GenBank (PQ724474.1) per the Centers for Disease Control and Prevention (CDC) (14). This sequence is named A/California/192/2024.

To generate a phylogenetic tree, H5 gene sequences from human cases in North America were identified from the GISAID database using the following search criteria: type, A; H, 5; N, any; host, human; location, North America; collection dates, 1 January 2024 to 30 April 2025. A total of 63 sequences were obtained, including this case, and were aligned using Clustal X 2.1. All sequences were trimmed to match the boundaries of A/California/192/2024. We excluded sequences that did not cover the complete 581-base region sequenced in this case, as well as duplicate sequences from serial passages of the same isolates and sequences from cases lacking clear documentation of the type of animal exposure. Multiple sequence alignment was performed on the remaining 46 sequences using Clustal X 2.1 with the option “ignore gaps.” A phylogenetic tree using the neighbor-joining method was generated in Clustal X 2.1 and then rendered in Mega 11 with options “root at midpoint” and “arrange for balanced shape.”

## RESULTS

A total of 4,562 influenza A-positive nasopharyngeal swab samples collected within the Stanford Health Care system between 7 August 2024 and 12 April 2025 were reflexed to H5 subtyping. Of these, the pan-influenza A target was detected with a C_T_ value of less than 40 cycles in 98.4% (4,488/4,562) of the samples, with a median C_T_ value of 21.7 cycles (interquartile range 18.2–26.7). Of the remainder, there were 25 samples in which the pan-influenza A target C_T_ value was detected but exceeded 40 cycles, 42 samples in which the pan-influenza A target was not detected and the internal control was detected, and 7 samples in which both the pan-influenza A target and internal control were not detected. H5 was detected in two samples, resulting in a positivity rate of 0.04% (2/4,488).

### Case 1: 14-month-old girl

A 14-month-old girl presented with her parent to an urgent care facility for evaluation of fever, congestion, and behaving “fussier than usual.” The patient had not consumed unpasteurized milk and did not have exposures to dairy farms or to sick animals, including cows, birds, or domestic pets. The patient had exposure to sick children at daycare, as well as to family members with similar symptoms.

The patient had a heart rate of 114 beats/min, respirations of 28 breaths/min, oxygen saturation of 98% (room air), and a rectal temperature of 36.8°C. The child was noted to be well appearing and not in distress. Conjunctiva and sclera were clear bilaterally. No rhinorrhea was noted, and the oropharynx was normal. Both tympanic membranes were erythematous with effusions bilaterally, consistent with acute otitis media, originally diagnosed in the prior month. The respiratory examination was unremarkable. The patient was discharged home on a 7-day course of oral amoxicillin (90 mg/kg/day).

A throat swab was negative for Group A *Streptococcus* by PCR. However, a nasopharyngeal swab was positive for influenza A using a 4-plex (Influenza A/B/RSV/SARS-CoV-2) respiratory virus RT-PCR (Cepheid, Sunnyvale, CA). The patient was started on a 5-day course of oseltamivir the following day.

**Fig 1:**
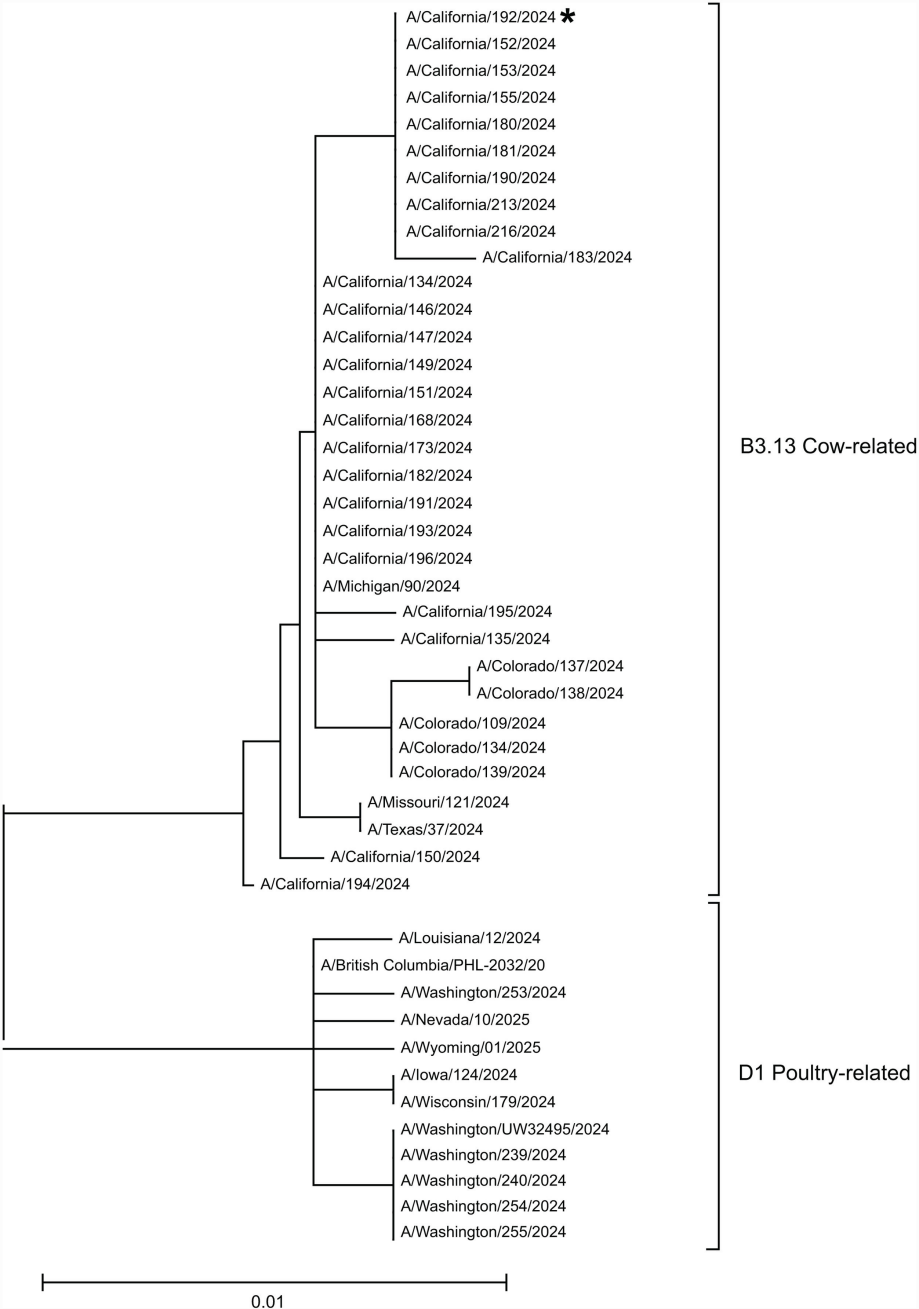
A phylogenetic tree of partial human H5 sequences identified in North America between January 2024 and April 2025 shows that the case in the 14-month-old girl (A/California/192/2024, GenBank PQ724474.1, labeled with an asterisk) is closely related to other cases with the dairy cow-associated B3.13 genotype.

This patient’s sample was positive for H5 with a C_T_ value of 34.0; pan-influenza A was also positive with a C_T_ value of 32.8. The sample was re-extracted and re-tested, and the H5 (C_T_ = 32.7) and pan-influenza A (C_T_ = 32.5) signals were reproducible. Further testing at the CADPH using the CDC Influenza A/H5 Subtyping Kit detected both the H5a and H5b targets (15). In-house partial sequencing of the HA gene revealed a sequence closely related to the H5 gene present in the clade 2.3.4.4b, genotype B3.13 influenza A(H5N1) viruses from California human cases (Fig. 1, GenBank PQ724474.1).

Retrospective testing using the BioFire Respiratory Panel 2.1 (bioMérieux, Marcy-l'Étoile, France) revealed that the original sample was equivocal for influenza A, indicating that one of two pan-influenza A assays was positive, and the H1, H3, and H1-2009 assays were negative. This pattern is typically observed with low levels of virus (16). The respiratory panel was also positive for adenovirus and rhinovirus/enterovirus. The child was re-evaluated 3 days after initial presentation and was well appearing with normal vital signs. Nasopharyngeal swabs collected from two of the patients’ family members were positive for adenovirus and rhinovirus and negative for influenza A.

### Case 2: 79-year-old male

A 79-year-old male with a history of hypertension, hyperlipidemia, type 2 diabetes mellitus, recurrent strokes, and dementia presented to an urgent care facility for evaluation of 1 week of cough and shortness of breath. The patient lives in a single-family home with a caregiver and had no known exposures. The patient was afebrile, with oxygen saturation of 96% (room air). Conjunctiva, sclera, as well as breath sounds were clear bilaterally. Chest X-ray showed bilateral lung opacities and peribronchial wall thickening. A nasopharyngeal swab was positive for influenza A using the 4-plex RT-PCR described above (Cepheid). The patient was prescribed oral cefpodoxime (200 mg twice a day for 7 days) and doxycycline (100 mg twice a day for 7 days) for community-acquired pneumonia and discharged home.

This patient’s sample was positive for H5 with a C_T_ value of 40.1; pan-influenza A was also positive with a C_T_ value of 38.1. The sample was re-extracted, and both the original and re-extracted eluates were re-tested. Both the H5 (re-extracted eluate, C_T_ = 37.3; original eluate, C_T_ = 35.8) and pan-influenza A (re-extracted eluate, C_T_ = 38.4; original eluate, C_T_ = 36.0) signals were reproducible. Further testing at the CADPH and CDC was negative for both pan-influenza A and the H5 subtype. There was insufficient residual sample or eluate for sequencing.

One day after discharge, the patient re-presented with a blood pressure of 70/40. His white blood cell count was elevated (18.2 K/µL), though he remained afebrile. An expectorated sputum was positive for *Escherichia coli* and human rhinovirus/enterovirus, but negative for influenza A, as well as 23 other pathogens using the BioFire Pneumonia Panel (bioMérieux). The patient was admitted and treated with intravenous fluids, ceftriaxone (intravenous, 2 g daily for 3 days), azithromycin (intravenous, 500 mg daily for 3 days), and oseltamivir (oral, 75 mg twice daily for 5 days). The patient’s condition improved, and he was discharged home 2 days after admission with a prescription for cefpodoxime (oral, 200 mg twice daily for 3 days).

## DISCUSSION

The lack of widespread H5N1 subtyping during the 2024 outbreak left open the possibility that animal-human spillovers, or human-to-human transmission events, were being missed by the public health surveillance system. However, the positivity rate of this universal H5 subtyping strategy in the San Francisco Bay Area during 2024–2025 was just 0.04%, identifying only two cases. These results confirm the lack of widespread H5N1 infection in low-risk individuals served by our healthcare system over this period. Nevertheless, detection of these outliers reveals possible blind spots in H5 surveillance which may lead to undetected outbreaks if further mammalian adaptation occurs (15).

These cases bear some similarities to other reported H5 cases without high-risk exposures; in particular, both our Case 1 and another case in San Francisco occurred in young children with mild symptoms and were found to contain virus related to the virus B3.13 genotype, which has circulated in U.S. dairy cows (6). In contrast, two severe cases were caused by genotype D1.1: one in British Columbia, Canada, and another in Louisiana (1).

However, it remains unclear whether the cases reported here reflect true infections. According to current CDC case definitions for novel influenza A infections, Case 1 meets criteria for a “confirmed” case, while Case 2 does not strictly meet criteria for the “confirmed,” “probable,” or “suspect” definitions (17). Neither of the patients exhibited symptoms typically associated with occupational exposure in the recent dairy-associated outbreak, such as conjunctivitis, and both experienced relatively mild symptoms with rapid recovery. Furthermore, neither patient had exposure to known human cases of H5N1 or sick animals or birds. While the RT-PCR results were reproducible and there was no laboratory evidence of contamination, the high cycle threshold values indicate very low levels of viral nucleic acids. This low-level detection is reflected in the inability of external laboratories to confirm the case in the 79-year-old male, which may have been the result of differences in assay sensitivity or RNA degradation (for example, due to additional freeze-thaws). Both cases were also found to be positive for other respiratory pathogens that could explain their clinical presentations. Studies have demonstrated that a substantial portion of the commercial milk supply contains H5 nucleic acids (18); therefore, we hypothesize that these patients may have consumed milk containing H5 RNA, which was detected in their nasopharynx at the time of sampling.

The strengths and weaknesses of a universal surveillance strategy merit careful consideration. The financial and logistical costs of increased testing must be weighed against the consequences of missing potential cryptic transmission and the benefit of early detection to allow intervention before an outbreak develops into an epidemic or pandemic. Given the findings described here, it may be beneficial to expand H5 subtyping beyond current testing criteria, with a focus on geographic areas with higher risk of zoonotic spillover. Notably, the positive cases we identified resided in an area that is adjacent to counties with high-density dairy farming operations. Thus, expanded subtyping may prove beneficial if targeted toward influenza A-positive samples collected from patients residing in zip codes within or more proximal to higher-risk regions (for example, California’s Central Valley). Innovations, including testing of pooled samples, may increase testing capacity while decreasing costs associated with additional surveillance.

In addition to expanded screening of clinical samples, implementing complementary strategies, including systematic testing of the commercial milk supply, targeted serological surveys, wastewater surveillance, and genomic monitoring for mutations of concern, may provide comprehensive, critical early warning for potential pandemic H5 threats. These approaches would help to shift our posture from pandemic response to pandemic prevention through early detection and targeted control measures.

## Data Availability

All information necessary to evaluate the findings of the paper is included in the paper. Additional data may be provided by the authors upon request. Further information and requests should be directed to the lead contact, B.A.P.
